# Evaluation of disease burden and response to treatment in adults with type 1 gaucher disease using a validated disease severity scoring system (DS3)

**DOI:** 10.1186/s13023-015-0280-3

**Published:** 2015-05-22

**Authors:** Neal J. Weinreb, David N. Finegold, Eleanor Feingold, Zhen Zeng, Barry E. Rosenbloom, Suma P. Shankar, Dominick Amato

**Affiliations:** University Research Foundation for Lysosomal Storage Diseases Inc., 7367 Wexford Terrace, Boca Raton, FL USA; Childrens Hospital of Pittsburgh, One Children’s Hospital Drive, 4401 Penn Avenue, Pittsburgh, PA 15224 USA; University of Pittsburgh, 623 Parran Hall, 130 DeSoto Street, Pittsburgh, PA 15261 USA; Tower Cancer Research Foundation, 9090 Wilshire Blvd., Suite 350, Beverly Hills, CA 90211 USA; Emory University School of Medicine, 2165 North Decatur Rd, Atlanta, GA 30033 USA; Mount Sinai Hospital, 600 University Avenue, Toronto, ON M5G 1X5 Canada

**Keywords:** Gaucher disease, DS3, Severity Score, Genotype, Splenectomy, Gaucher disease therapy, Treatment responses, Health state transitions, Gaucher bone disease, Bone event-free survival

## Abstract

**Background:**

GD1-DS3 is an integrated assessment of type 1 Gaucher disease (GD1) burden based on bone, hematologic and visceral domains. We investigated this disease severity scoring system (DS3) methodology for initial assessment, long-term follow-up and evaluation of treatment responses.

**Methods:**

We enrolled 133 treated adult GD1 patients. Baseline DS3 scores were calculated near the initial treatment date and patients stratified by severity as marked (DS3 6.00-19.00), moderate (DS3 3.00-5.99), mild (DS3 < 3.00). Follow-up scores were calculated annually. Minimal clinically important improvement (MCII), is defined as ΔDS3 of -3.1.

**Results:**

*Patient characteristics*: N370S was the most common allele (118 patients had at least one), 52 were N370S/N370S (48/52 were Ashkenazi Jews), N370S/L444P was the most common genotype among non-Jews. Median age of treatment: 45 years; median follow-up: 14 years. *Baseline DS3 scores:* Patients with marked disease (*N* = 58; median 7.84) were least likely to be N370S homozygous (19 %) and most likely to have had splenectomy (53 %), early age at diagnosis (median 18 years) and major pre-treatment bone pathology (76 %). Among patients with moderate disease (*N* = 53; median 4.33), 49 % were N370S/N370S, 15.1 % had splenectomy and 17 % had major bone disease. Median age at diagnosis: 32 years. No patient with mild disease (*N* = 22; median 2.4) had splenectomy or major skeletal disease. Median age at diagnosis: 40 years. 68 % were N370S homozygous. *Response to treatment*: Health-state transitions occurred primarily during the early treatment years. At Year 5, among 48 evaluable patients with marked baseline disease, eight were unchanged in severity status whereas 40 had MCII of varying degrees with 11 scored as mild. Among 42 evaluable moderate patients, none worsened, 16 remained moderate and 26 improved to mild. Among 16 evaluable mild patients, 14 remained so and 2 had DS3 scores in the low moderate range.

**Conclusions:**

DS3 is effective for assessing disease burden in GD1 and for monitoring response. ERT was associated with MCII in DS3 scores in patients with high severity. Nevertheless, despite better DS3 scores with treatment, GD1 patients especially those with splenectomy and pre-treatment bone pathology, continued to have bone complications.

## Background

Gaucher disease Type 1 (GD1, OMIM 230800), a recessively inherited, pan-ethnic glycosphingolipid storage disorder is caused by deficient activity of lysosomal acid β-glucosidase (glucocerebrosidase, EC3.2.1.45, GCase) resulting from pathogenic variations in the GCase gene, GBA1 [1]. GD1 is one of the most prevalent LSDs and the first to be successfully treated with pharmacologic recombinant enzyme replacement therapy (ERT) [2]. Disease expression is diverse, subject to genotype, other genetic modifiers [3, 4] and yet undefined epigenetic and environmental factors. Untreated patients with GD1 may be asymptomatic with few signs of disease or present with combinations of hematologic abnormalities, hepatosplenomegaly, pulmonary involvement and a spectrum of skeletal pathologies that may cause substantial morbidity, functional disability and decreased health-related quality of life [1]. Signs and symptoms may occur anytime from early childhood to late adulthood. The rate and extent of disease progression is variable, unpredictable, and often independent of the age at which disease manifestations are first detected [1]. Significant heterogeneity in rate of improvement in hematologic, visceral and bone manifestations exists in treated patients [5, 6]. There are few well-designed studies that have comprehensively annotated phenotypic variation over time or measured treatment efficacy and dose response [7, 8]. Design of such studies was partly hindered by lack of a validated disease severity scoring system for GD1 to standardize the monitoring of progression and treatment response and to define patient cohorts in clinical studies [9]. Early disease severity scoring indices such as Zimran score [10] and Hermann score for bone disease [11] primarily emphasized advanced and irreversible disease manifestations. These scores also had limited sensitivity for annotating changes observed over time in untreated patients with slow progression or in treated patients with irreversible manifestations. A newer system, Gaucher Disease Severity Score Index–Type 1 (GAU-SSI-1), is complex and requires specialized technology [12]. Other models for monitoring treatment response focused on composite achievement of therapeutic goals that are assigned equal weight without attention to relative clinical import [5].

A disease severity scoring system (DS3) is a method of expressing an integrated assessment of disease burden. It may be used to monitor patient status, determine endpoints in clinical studies, classify disease phenotypes and compare patients with the same disease. Although often called ‘disease severity indices’, DS3s may also include measures of disease activity and organ damage. They are usually structured as a group of domains (often according to organ system) populated with non-redundant items that are valid, reliable, use feasible and standardized methods of assessment, and are variably weighted based on associated morbidity and mortality [13]. In 2010, Weinreb et al. reported preliminary testing for validity, reliability and feasibility of a DS3 for adult patients with GD1 [14]. When 20 patient profiles from the International Collaborative Gaucher Group (ICGG) Gaucher Registry [15] were independently evaluated and scored by 12 expert GD1 physicians, the GD1-DS3 correlated very well with a “gold standard” clinical global impression scale. An additional panel of 23 physicians with GD1 expertise evaluated the DS3 instrument for reliability, feasibility and construct, criterion and content validity. Inter-rater reliability was high (0.97, Cohen’s kappa). The feasibility and content validity indices were also high (0.95 and 0.96, respectively) [14]. However, expanded testing in a more diverse population including splenectomized patients has not been completed. Here, we further investigate the DS3 score as a tool for initial assessment and evaluation of treatment responses in GD1 patients within a consortium of five geographically separated North American GD treatment centers.

## Methods

### Patient population

Eligibility criteria included men and women with GD1 ≥ 18 years old at time of recruitment, regardless of race, ethnicity or treatment status. Subjects who started treatment as children were eligible, but data entry was restricted to dates after the 18th birthday. Diagnosis of GD was confirmed by leukocyte or skin fibroblast acid β-glucosidase deficiency and/or DNA genotype analysis. Eligibility was also contingent on enrollment in the ICGG Registry. Neuronopathic GD (GD3) was ruled out based on combinations of personal and family history, physical examination, and genotype analysis.

Patients were recruited at University Research Foundation for Lysosomal Storage Diseases, Coral Springs, Florida; University of Pittsburgh, Pittsburgh, Pennsylvania; Tower Cancer Research Foundation, Beverly Hills, California; Emory University, Atlanta, Georgia; and Mount Sinai Hospital, Toronto, Canada. All participating patients who were alive prior to the inception of the DS3 study signed separate informed consents to participate in the DS3 Severity Score Study and the ICGG Registry using forms approved by the respective institutional review boards. Data from 13 patients who died prior to 2010 were included based on previously signed ICGG Registry consent forms. An ICGG-independent data center calculated the DS3 scores and collated the phenotypic information. The DS3 Severity Score Study is registered on Clinicaltrials.gov (NCT01136304).

### Genotype

Genotypes were reported by the participating investigators. In most instances, genotypes were determined by PCR specific oligonucleotide screening for common mutations prevalent in North America (e.g., N370S, L444P, 84GG, IVS^2 + 1^, R496H, V394L). Samples from some patients with unidentified alleles were referred to either the ICGG Registry Genotyping Service at the laboratory of H. Ronald Scott, MD, PhD at the University of Washington, Seattle, WA, USA or the laboratory of Francis Choy, Victoria, BC, Canada, for whole gene sequencing.

### Gaucher disease severity scoring

**Fig. 1:**
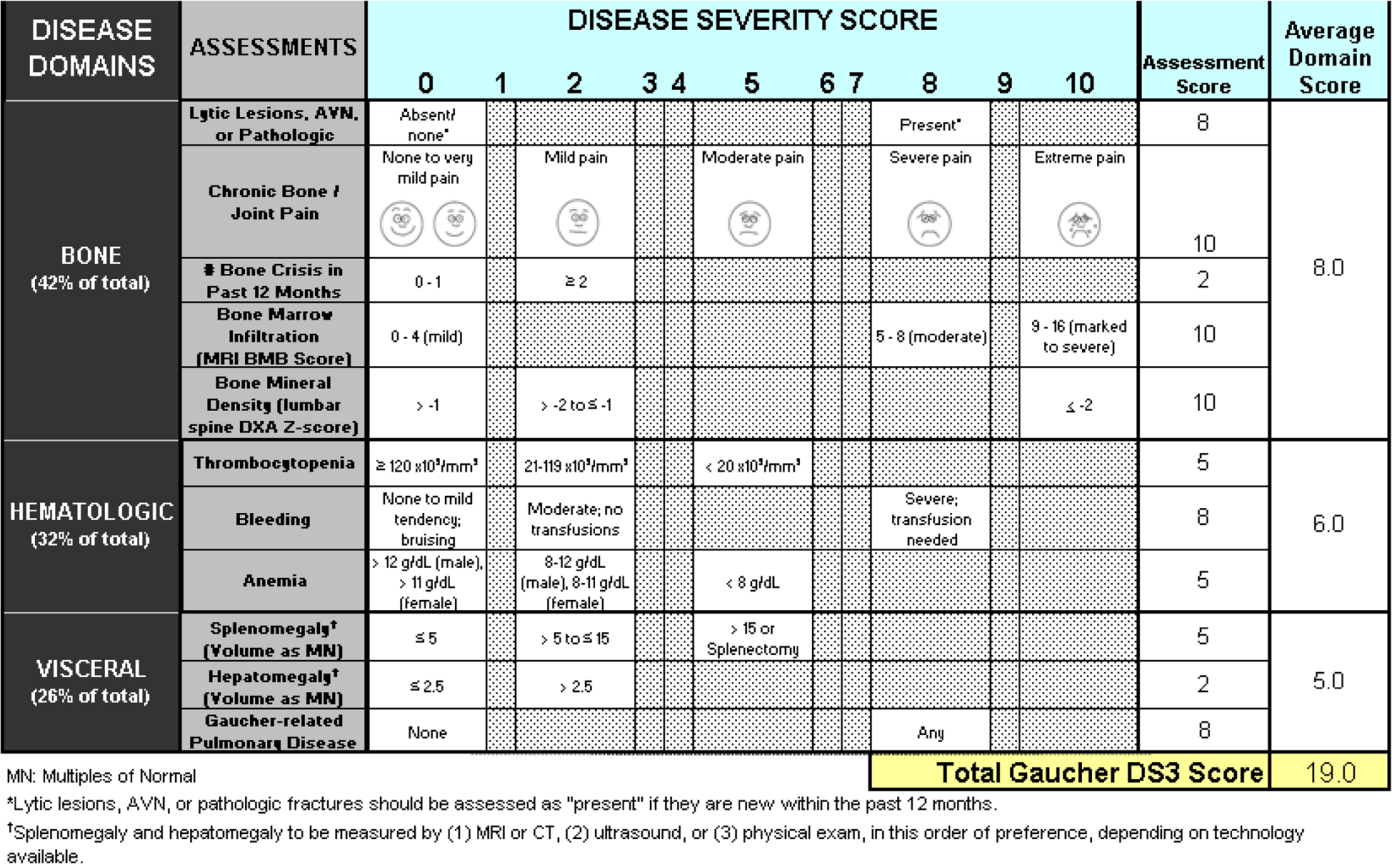
Gaucher Disease Type 1 Severity Scoring System (GD-DS3)

DS3 severity scoring was performed retrospectively based on data entries in the ICGG Registry database supplemented by the study DS3 database according to instructions in Fig. 1 and evaluated per instructions in Table 1. Because of irreversible bone and joint damage and frequent concurrent causes for musculoskeletal pain especially in older GD1 patients with Registry data entered by multiple, variably experienced observers over long periods of time, we suspect that the instruction in Table 1 to restrict evaluation of bone pain to GD-specific etiologies was often not accomplished in this retrospective study and possibly unrealistic even in prospective studies and clinical trials. Disease severity states were originally defined [14] as:

**Table 1:** Gaucher disease type 1 DS3 scoring

General instructions	
	1. Record date of assessment
	2. For each assessment, determine the GD1-DS3 score of the patient at the time of evaluation (See notes below regarding specific assessments).
	a. If current data are not available for all assessments when the DS3 score is calculated, data from previous evaluations may be used if the patient’s overall clinical status has remained stable and assessments (bone and visceral imaging, hematological) were collected within 12-24 months before the current date:
	b. If bone marrow infiltration and/or bone mineral density data are not available at the time of assessment or from previous evaluations, the DS3 has been optimized to be accurate and consistent without these parameters.
	c. All other assessment scores within the time frames described above are required.
DS3 score calculation	
	1. First calculate the average Disease Domain Scores by adding the assessment scores for each domain (bone, hematological, visceral) and dividing by the number of assessment scores completed. Do not include assessments that were marked “not available” (NA)
	2. The total GD1-DS3 score is the sum of the three Disease Domain Scores.
Maximum possible DS3 score	
	1. The maximum possible DS3 score is 19.
	2. In initial validation testing using 20 patient cases scored at 2 different time points, no patient received a score higher than 13 and scores above 9 correlated with an expert assessment of “severe disease.”
Interpretation of GD1-DS3 scores	
	0-3 Borderline to mild disease
	3-6 Moderate disease
	6-9 Marked disease
	>9 Severe disease
Notes regarding specific assessments	
	1. Lytic lesions, AVN or pathologic fracture “present” means any new occurrence in the past 12 months
	2. Bone marrow infiltration may be reported either semi-quantitatively (BMB score) or qualitatively (mild, moderate, marked to severe
	3. For bleeding, an assessment of moderate (no transfusions) or severe (transfusion needed) should be based on bleeding considered by the assessor to be related to GD, whether due to low platelet count, other hemostatic disorders or vascular disease such as portal hypertension
	4. Assessment of bone pain should be based on severity in the absence of analgesics and should consider only pain resulting from GD rather than pain attributable to other concurrent musculoskeletal diseases.

- Severe (DS3 9.00-19.00)
- Marked (DS3 6.00-8.99)
- Moderate (DS3 3.00-5.99)
- Mild (DS3 < 3.00)

In Weinreb et al. [14], no test patient had a DS3 score greater than 12.0. Of five patients with DS3 9.0-12.0, three were classified by the panel of 12 GD experts as having “severe” disease and two were evaluated as having “marked” disease. Comparable variation in the clinical global expression “gold standard” scale was not observed for the marked, moderate or mild categories suggesting that a clinical distinction between marked and severe disease may be unclear. For this reason, and because only 15 of our study patients had DS3 scores ≥ 9.00 (and only 6 greater than 9.33), we decided to conflate the “marked” and “severe” categories into one “marked” disease state defined as DS3 9.00-19.00.

In reporting treatment outcomes, the minimal clinically important difference (MCID) is often more meaningful than the minimal detectable, statistically significant difference [16]. MCID is the smallest change that a patient or a clinician would identify as sufficiently important so as to justify a change in management or treatment plan. MCID may be determined by distribution based (statistical) methods, by patient-oriented anchor methods, or by expert based nominal group technique (Delphi) methods. There is no consensus on which method is best [17]. The MCID for DS3 was determined by Delphi method as described in Weinreb et al. [14]. Because no patient in this study met the DS3 MCID criterion for clinical deterioration, in this report, we refer solely to minimal clinically important improvement (MCII).

Eligibility for the study was not restricted by treatment. 132/133 enrolled patients initially received ERT. One patient (baseline DS3 5.53) was treated solely with miglustat for three follow up years. Because scientific questions related to untreated patients primarily concern natural rather than modified evolution of disease, we chose to defer analysis of untreated patients to a later study. Baseline DS3 scores were calculated just prior to or at the time of initiation of treatment. Follow-up scores were calculated annually with a ±3 month window. Registry data entry usually post-dated the occurrence of bone complications such as avascular necrosis (AVN) and fractures. Retrospectively, we were unable to correlate timing of bone events with acute symptoms such as bone pain. In routine clinical practice, MRI evaluation of bone marrow infiltration and measurement of bone mineral density are rarely performed annually. Because the calculation of DS3 scores is sensitive to missing data for these parameters, some entries for bone marrow infiltration and bone mineral density DS3 component scores were imputed as unchanged when data for the preceding and subsequent year were identical. When imputation was not possible, scores were *per force* computed without the missing parameters as described in Table 1.

### Statistical analyses

Two-tailed *t*-test was used for comparing the mean of continuous outcomes between two groups. Fisher’s exact test was used to check the independence among multiple groups in contingency tables. The significance test for correlations was the *t*-test from corresponding simple linear regressions. Disease progression among groups was compared using Kaplan-Meier survival estimates. The equivalence of survival curves among difference groups was examined using the log-rank test. All the analyses were performed in R, version 3.0.3 (The R Project for Statistical Computing).

## Results

### Data quality and completeness

Due to our ability to supplement ICGG Registry data with primary source data at the study sites, missing data was less problematical than we anticipated. The demographic and pre-treatment characteristics data sets were essentially complete as shown in the relevant tables below. Information about ethnicity and GBA1 genotype was unavailable for 8 and 6 of the 133 treated study patients.

Data needed to calculate annual DS3 scores was less easy to recover although often sufficient to allow imputation. Data was imputed for 465 of 1415 possible bone marrow infiltration entry lines (32.9 %), for 30.2 % of DEXA BMD entries, for 26.9 % of spleen volume entries and for 42.6 % of liver volume entries. The difference between liver and spleen is attributed to patients with a history of splenectomy. The imputation rate for bone pain was 5.8 %, for major bone lesions, 2.6 %, and ≤ 1.0 % for bone crises, anemia, thrombocytopenia, clinical bleeding and pulmonary involvement. The amount of missing data that could not be imputed was less than 1.0 % for all DS3 components except bone marrow infiltration (4.4 %) and DEXA BMD (31.0 %). Non-imputable, missing DEXA data was largely from the 1990’s and early 2000’s when BMD measurement had not yet been included in the recommended evaluations for GD1. We believe that missing data does not significantly impact the validity of the results we herein report.

### Patient enrollment and demographics

One hundred seventy three patients were recruited. Seven patients were excluded because data was insufficient to calculate baseline DS3 scores and relevant medical records were not available for review. Of 166 included patients, 33 were untreated. 133 patients started either ERT (*N* = 132) or miglustat (*N* = 1) a median 14 years (range 1-23 years) prior to the analysis date.

61 % of the patients were female with a majority of women at all sites except Los Angeles (Table 2). 68 patients (51.1 %) identified all four grandparents as Ashkenazi Jews. An additional 13 (9.8 %) claimed partial Ashkenazi Jewish descent. The majority of patients from South Florida and Los Angeles were Jewish, whereas non-Jewish patients constituted the majority in Toronto, Atlanta and Pittsburgh. The median age (range) at the time of GD1 diagnosis was 28 (0-85) years. One-third of the patients were diagnosed before 18 years of age. GD1 treatment was begun at a median age of 45 (6-87) years. The median interval between diagnosis and treatment was 17 years. Before 1991 (when alglucerase was approved in the US), the interval between diagnosis and treatment was 2 years or less in only 1 of 79 patients (1.3 %) and greater than 5 years in 74 (93.7 %). Among 54 patients diagnosed in 1991 or later, the median interval between diagnosis and start of treatment was 2 years. The interval between diagnosis and treatment was greater than 5 years in only 6 patients (11.1 %).

**Table 2:** Baseline (pre-treatment) characteristics of 133 treated patients with GD1 by investigative site

	Total	South Florida	Toronto	Atlanta	Los Angeles	Pittsburgh
Patients: N	133	61	25	15	24	8
Patients with splenectomy: n (%)	39 (29.3)	17 (27.9)	8 (32.0)	5 (33.3)	5 (20.8)	4 (50.0)
Women: n (%)	81 (61)	36 (59)	18 (72)	10 (66.7)	11 (45.8)	6 (75)
Ethnicity: n (%)						
Ashkenazi Jewish	68 (51.1)	45 (73.8)	4 (16.0)	3 (20.0)	15 (62.5)	1 (12.5)
Part Ashkenazi	13 (9.8)	7 (11.5)	1 (4.0)	1 (6.7)	4 (16.7)	0
Non-Jewish	44 (33.1)	9 (14.8)	19 (76.0)	8 (53.3)	2 (8.3)	6 (75.0)
Unknown	8 (6.0)	0	1 (4.0)	3 (20.0)	3 (12.5)	1 (12.5)
Age at diagnosis (y)						
Mean (SD)	29.7 (18.8)	34.6 (21.2)	17.0 (19.2)	30.7 (14.1)	32.0 (18.4)	23.4 (19.1)
Age at first treatment (y)						
Mean (SD)	44.5 (18.8	52.2 (16.9)	33.5 (19.3)	37.3 (17.2)	43.1 (16.3)	38.5 (19.0)
Years on treatment						
Mean (SD)	13.3 (6.1)	13.3 (6.0)	12.0 (5.6)	14.3 (6.4)	12.8 (6.8)	17.0 (4.6)
Current age (y)						
Mean (SD)	57.8 (18.4)	65.5 (15.5)	45.5 (18.9)	51.6 (18.8)	55.9 (16.5)	55.5 (19.2)
Genotype: N (%)						
N370S/N370S	52 (39.1)	35 (57.4)	3 (12.0)	5 (33.3)	8 (33.3)	1 (12.5)
N370S/L444P	24 (18.1)	8 (13.1)	7 (28.0)	5 (33.3)	2 (8.3)	2 (25.0)
N370S/84GG	7 (5.3)	4 (6.6)	2 (8.0)	0	1 (4.2)	0
N370S/IVS^2+1^	4 (3.0)	3 (4.9)	0	0	0	1 (12.5)
N370S/other	31 (23.3)	9 (14.8)	10 (40.0)	4 (26.7)	7 (29.2)	1 (12.5)
L444P/other	3 (2.2)	1 (1.6)	1 (4.0)	0	0	1 (12.5)
L444P/L444P	1 (0.75)	0	1 (4.0)	0	0	0
Other/other	5 (3.8)	1 (1.6)	1 (4.0)	1 (6.7)	0	2 (25.0)
Unknown/missing	6 (4.5)	0	0	0	6 (25.0)	0
Baseline DS3 score*						
Mean (SD)	5.6 (2.6)	5.6 (2.5)	6.6 (2.9)	4.4 (2.3)	4.8 (2.4)	7.2 (2.0)

*Two-tailed t-test: Toronto v Atlanta: P=0.016; Toronto v California: P=0.020; Pittsburgh v Atlanta: P=0.008; Pittsburgh v California: P=0.016

At initiation of treatment, 55 of 126 patients (44 %) had one or more co-morbidities that apparently were unrelated to GD. Pre-treatment total splenectomy was reported in 39 of 133 patients (29.3 %) with little inter-site variation. The median age at splenectomy was 25 years (Range: 3-73 years; Interquartile range: 7.5-36 years). Eight (20.5 %) patients had splenectomy before age 6 years, 5 (12.8 %) between 6 and 18 years and 26 (66.7 %) after age 18 years. Among patients diagnosed prior to 1991, 36 of 79 (45.6 %) had a splenectomy before starting treatment but only 3 of 54 (5.6 %) diagnosed in 1991 or later (the ERT era) were splenectomized. 43 of 79 patients diagnosed before 1991 (54.4 %) had a history of a major bone event (AVN, fracture, or lytic lesion) prior to starting treatment compared to 10 of 54 (18.5 %) of those whose year of diagnosis was 1991 or later. Two of the 10 patients (20 %) diagnosed post-1991 with pre-treatment bone events had a history of splenectomy compared to 26 of 43 diagnosed before 1991 (60.5 %).

### Genotypes

Due in part to ethnic differences, substantial variation in genotype distribution was observed among the investigative sites (Table 2). N370S homozygosity was especially prevalent in South Florida and uncommon in Toronto and Pittsburgh. 88.8 % of the 133 patients had at least one N370S allele. 48 of 52 N370S homozygous patients had complete or partial Ashkenazi Jewish ethnicity. N370S/L444P was the single most common genotype among non-Jewish patients but was also found in 6 of 86 (8.8 %) Ashkenazi Jewish patients. Two self-identified non-Jewish patients (N370S/84GG, N370S /IVS^2+1^) may have had incorrect or incomplete information about their ethnic background.

Qualitative genotype-phenotype correlations were observed for clinical phenomena that are traditionally invoked as markers of disease severity (Table 3). Pre-treatment splenectomy was significantly less common and age at diagnosis was significantly greater in patients homozygous for N370S than in the other genotype groups. Additionally, the lowest percentage of patients with pre-treatment severe bone events was found among homozygous N370S patients.

**Table 3:** Splenectomy status, mean age at diagnosis, and pre-treatment bone complications (avascular necrosis, fractures, lytic lesions) and mean baseline DS3 score by genotype categories

Genotype (N)	Splenectomy*	Pre-treatment* bone events	Mean (SD) age at diagnosis (y)	Mean (SD) baseline DS3 score
N370S/N370S (52)	6 (11.5 %)^€^	12 (23.1 %)	42 (17)	4.33 (2.30)
N370S/L444P (24)	7 (29.2 %)^€^	8 (33.3 %)	22 (15)^§^	6.08 (2.49)^§^
N370S/other (42)	19 (45.2 %)^€^	25 (59.5 %)	20 (18)^§^	6.68 (2.47)^§^
Other/other (9)	7 (77.8 %)^€^	6 (66.7 %)	20 (20)^§^	7.17 (2.32)^§^
Unknown (6)	0	2 (33.3 %)	36 (28)	4.77 (1.57)

*Of 53 patients with pre-treatment bone events, 25 (47.1 %) had intact spleens and 28 (52.9 %) had splenectomies. Of the 12 N370s homozygotes with pre-treatment bone events, 8 had intact spleens and 4 had splenectomy

^€^Splenectomy probabilities: Exact test: *P* = 0.0082, including unknown patients; *P* = 0.0074, excluding unknown patients

^§^Two-tailed *t*-test

Mean baseline DS3 score

Mean age at diagnosis

N370S/N370S v N370S/L444P: *P* = 0.0041

N370S/N370S v N370S/L444P: P < 0.0001

N370S/N370S v N370S/other: P < 0.0001

N370S/N370S v N370S/other: P < 0.0001

N370S/N370S v other/other: *P* = 0.0012

N370S/N370S v N370S/other: *P* = 0.0009

### Baseline DS3 severity scores

The mean (SD) baseline DS3 severity score for all 133 patients was 5.6 (2.6) with a 5.5 median score (0.4-14.9; inter-quartile range 3.7-7.5). The mean baseline DS3 scores varied among investigative sites but no site was significantly different from South Florida (Table 2). Mean DS3 scores were highest at the centers with the least number of Ashkenazi Jews.

Baseline mean DS3 scores differed by genotype and were lowest (in the moderate range) among the 52 N370S homozygous patients. Mean DS3 scores were in the marked severity range for all other known genotype sub-groups (highest in the other/other category). Pre-treatment DS3 scores were in the moderate or marked range in 71.2 % of the N370S homozygous patients, and, although uncommon, baseline DS3 scores in the mild range were found for patients with N370S/L444P and N370S/other genotypes (Table 4). The mean (SD) baseline DS3 score in 39 patients with a history of pre-treatment splenectomy was 7.80 (2.05) vs. 4.69 (2.21) in 94 patients with intact spleens (P < 0.0001). Of 53 patients with “moderate” DS3 severity, 7 had a history of splenectomy. Only one of these patients was younger than 18 years old at the time of splenectomy. Of 58 patients with “marked” DS3 severity, 31 had a history of splenectomy of whom 12 (38.7 %) were younger than 18 years when splenectomy was performed. In 53 patients with a history of pre-treatment severe bone events, the mean (SD) baseline DS3 score was 7.61 (1.77) vs. 4.29 (2.20) in 76 patients with no such history (P < 0.0001). Pre-treatment bone history was unknown in 4 patients. The mean (SD) baseline DS3 score in 45 patients diagnosed with GD1 when younger than 18 years was 6.70 (2.24) vs. 5.04 (2.58) in 88 patients older than age 18 years (*P* = 0.0004). For 79 patients who were diagnosed earlier than 1991, the mean (SD) baseline DS3 score was 6.54 (2.44) vs. 4.23 (2.16) for 54 patients diagnosed with GD1 in 1991 or thereafter (P < 0.0001).

**Table 4:** Characteristics of treatment response sub-groups defined by baseline DS3 score (mild: 0-0.299, moderate: 3.00-5.99, marked:* 6.00-19.00)

Characteristics	Mild (*n* = 22)	Moderate (*n* = 53)	Marked (*n* = 58)
Baseline DS3 (mean (SD)*	2.03 (0.82)	4.43 (0.92)	8.03 (1.49)
Splenectomy: N (%)	0	8 (15.1)^§^	31 (53.4)^§§^
Age (y) at diagnosis			
Median (Range)	40.5 (1-71)	32.0 (4-85)	18.5 (0-68)
Age (y) at 1^st^ treatment			
Median (Range)	44.5 (9-73)	50.0 (6-87)	44.5 (7-69)
Genotype**			
N370S/N370S (*N* = 52)	15 (28.8 %)	26 (50.0 %)	11 (21.2 %)
N370S/L444P (*N* = 24)	3 (12.5 %)	8 (33.3 %)	13 (54.2 %)
N370S/other (*N* = 42)	3 (7.1 %)	13 (31.0 %)	26 (61.9 %)
Other/Other (*N* = 9)	0	2 (22.2 %)	7 (77.8 %)
Unknown (*N* = 6)	1 (16.7 %)	4 (66.6 %)	1 (16.7 %)

*DS3 scores >10.0 were found in only 3 patients and no baseline DS3 score exceeded 14.9. Mean DS3 score (two-tailed *t*-test): Marked v Moderate: P < 0.0001; Mild v Moderate: P < 0.0001

^§^The DS3 score exceeded 4.33 in 7 of the 8 patients

^§§^20 of 29 patients (69.0 %) with a baseline DS3 ≥ 8.00

**Percentages refer to the genotype category

As shown in Table 4, patients were categorized as having marked, moderate or mild GD1 severity based on pre-treatment DS3 scores. Patients with marked severity were much more likely to have a history of splenectomy and a substantially younger age at GD1 diagnosis than those in the moderate or mild categories. However, the three groups differed very little regarding the age at which treatment was initiated. For the entire 133 patient cohort, a negative correlation exists between age at diagnosis and baseline DS3 score (*ρ* = -0.33, *P* = 0.0001), and a positive correlation between years from diagnosis to start of ERT and baseline DS3 score (*ρ* = 0.34, P < 0.0001). No correlation exists between age at which treatment was begun and the baseline DS3 score. Within each severity category, there was no correlation between baseline DS3 score and either age at diagnosis or age at start of ERT.

### Changes in DS3 scores following initiation of ERT or miglustat

Initially, 57 patients received alglucerase, 73 imiglucerase, 2 velaglucerase, and 1 miglustat. At the most recent follow-up, no patient was receiving alglucerase. 71 patients were receiving imiglucerase, 33 velaglucerase, 1 taliglucerase, 6 miglustat, and 6 were on clinical trials with eliglustat tartrate. Treatment had been interrupted in 15 patients and, in one patient, treatment status is unknown. The median (range) starting ERT dose was approximately 60 (8-60) units/kg/every 2 weeks (Q2W) and did not vary significantly based on the DS3 severity score (Table 5). Regardless of ERT product, the median (range) ERT dose at the time of most recent follow-up continued to approximate 45 (10-120) units/kg/Q2W. However, the dose tended to be somewhat higher in patients categorized as having marked disease severity based on pre-treatment DS3 score. For these response analyses, we have assumed that all commercial ERTs are bio-similar in efficacy and are freely interchangeable, although that is not yet definitively proven [18].

**Table 5:** ERT dosing per baseline DS3 severity score category (marked, moderate, mild) when treatment was initiated and at time of last follow up

	ERT dose at initiation of treatment (units/kg/Q2W)			ERT dose at time of last follow-up (units/kg/Q2W)		
DS3 category	No. of pts	Median	Range	No. of pts	Median	Range
Marked	58	60.0	8-60	47	50.0	15-120
Moderate	52	52.5	10-60	40	30.0	15-90
Mild	22	60.0	10-60	18	44.0	10-60

All ERT doses are expressed as units/kg body weight infused every 2 weeks (Q2W). For patients who were infused at intervals other than Q2W, the equivalent Q2W dose is shown. ERT dose calculations are inclusive of all ERT agents. At the time of last follow up (or death), 81 % of the initially marked severity patients continued to receive ERT, 77 % of the initially moderate severity patients continued to receive ERT, and 82 % of the initially mild severity patients continued to receive ERT

**Fig. 2:**
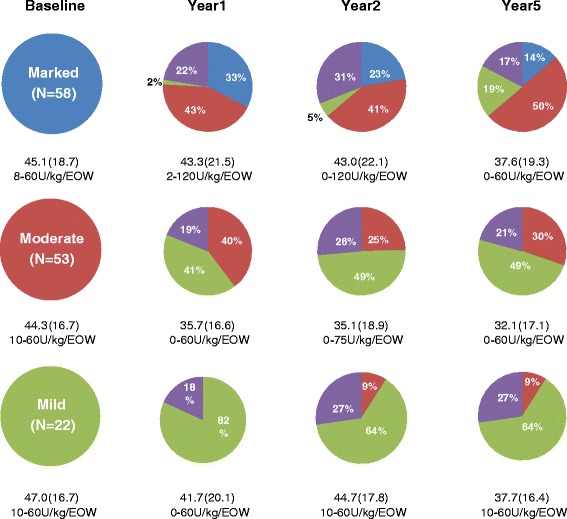
Categorical change from baseline DS3 severity status 1, 2, and 5 years after treatment initiated. Purple color indicates patients for whom data was unavailable for the year evaluated. Mean (SD) ERT doses and range in units/kg body weight/q 2 weeks are shown beneath the pie charts. Each sub-group includes a few patients in whom treatment was interrupted during an evaluation period and some patients were treated at very low doses

Responses to treatment were assessed longitudinally and as transitions between severity health states. Regardless of initial severity, significant changes from baseline DS3 scores occurred primarily during the early years of treatment. As shown in Fig. 2, among evaluable patients with initial marked severity disease, the proportion of patients in this category progressively decreased over 5 years. Most patients transitioned from marked to moderate, but 19 % improved to mild. Among evaluable initially moderate patients, 49 % were mild after 5 years of treatment and the others remained moderate. No patients progressed to marked disease. Most initially mild patients remained so throughout the 5 years.

Among evaluable patients with initial marked disease, the percentage of patients with minimal clinically important improvement (MCII) defined in the original GD1-DS3 manuscript as a -3.1 ΔDS3 from baseline [14] increased from 34.8 % after 1 year of ERT to 75.5 % after 5 years (Table 6). MCII occurred in 9 of 22 patients (40.9 %) in the moderate sub-group in whom pre-treatment DS3 scores ranged from 4.58-5.93, whereas no patients with baseline DS3 scores 3.00-4.50 had a MCII. No initially mild patient achieved MCII as the defined -3.1 Δ exceeds the maximum 2.99 score for this category. After correcting for initial DS3 severity score, there was no definite relationship between genotype and attained MCII status in patients with marked and moderate pre-treatment disease. MCII after 5 years of ERT occurred in 12 of 33 (36 %) of evaluable patients homozygous for N370S (baseline DS3 score 5.31) and in 32 of 56 (57 %) of evaluable patients with all other genotypes (baseline DS3 score 6.99). The likelihood of attaining MCII was minimally dependent on how quickly ERT was begun after GD1 diagnosis. Among 79 patients diagnosed prior to 1991 (in whom the interval between diagnosis and treatment exceeded 5 years in 74, 26 patients (33 %) had MCII after 5 years of ERT including 20 of 29 (69 %) with marked severity disease (DS3 ≥ 6.00). Among 54 patients diagnosed in 1991 or later (in whom the interval between diagnosis and treatment was less than 5 years in 48), 22 patients (41 %) had a MCII after 5 years of ERT including 17 of 21 (81 %) with marked severity disease.

**Table 6:** Cumulative ERT doses (units/kg) after 1, 2, and 5 years for GD1 patients with marked, moderate and mild DS3 baseline scores and treatment outcomes as determined by “minimal clinically important improvement” defined as a decrease of 3.1 from the baseline score for evaluable patients with marked and moderate baseline disease and 2.0 for patients with mild baseline disease

Baseline DS3 severity category	Number (%) of patients with clinically significant change in DS3 score			Mean (SD) cumulative ERT dose units/kg		
	Year 1	Year 2	Year 5	Year 1	Year 2	Year 5
Marked	16/46 (34.8)	19/40 (47.5)	37/49 (75.5)			
ΔDS3 ≥ 3.1	16	19	37	991 (447)	2354 (1136)	5681 (2277)
ΔDS3 ≤ 3.1	30	21	12	1249 (477)	2147 (857)	5163 (1775)
Moderate	6/42 (14.3)	6/39 (15.4)	9/44 (20.5)			
ΔDS3 ≥ 3.1	6	6	9	880 (238)	1835 (668)	3770 (1239)
ΔDS3 ≤ 3.1	36	33	35	1064 (391)	1854 (762)	4809 (2049)
Mild	2/17 (11.8)	0/16 (0.0)	2/16 (12.5)			
ΔDS3 ≥ 2.0	2	0	2	1170 (552)	NA	5850 (2758)
ΔDS3 ≤ 2.0	15	16	14	1131 (450)	2188 (925)	5478 (2493)

**Fig. 3:**
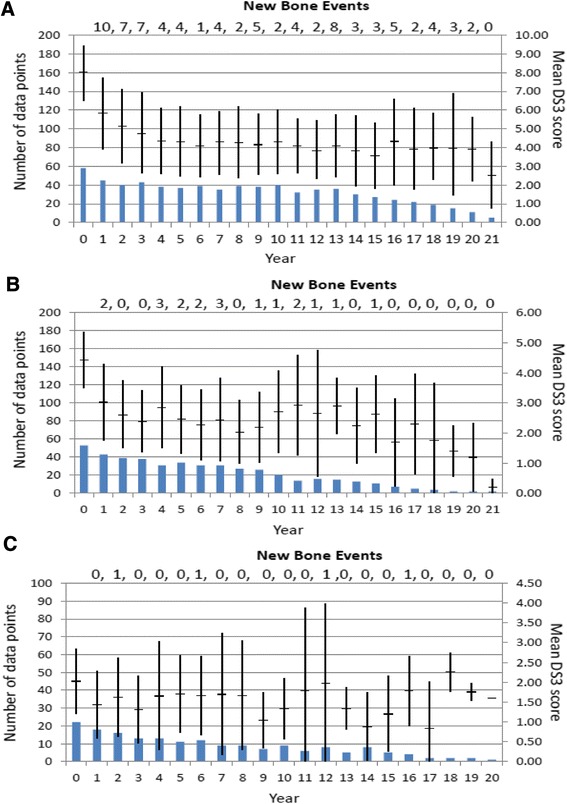
Changes in mean (± SD) DS3 score after initiation of GD1-specific treatment for patients with pre-treatment marked, moderate or mild disease as defined by baseline DS3 score **a** Baseline DS3 scores 6.00-14.87; **b** Baseline DS3 scores 3.00-5.93; **c** Baseline DS3 scores 0.40-2.93). Among 58 marked severity patients (baseline DS3 > 6), 38 patients (65.5 %) had a total of 82 new bone events (avascular necrosis, fracture, lytic lesions) while on treatment: 2.16 episodes per affected patient. Among 53 moderate severity patients (baseline DS3 3.00-5.99), 15 patients (28.3 %) had a total of 19 new bone events, 1.2 episodes per affected patient. Among 22 mild severity patients (baseline DS3 < 2.99), 4 patients (18.2 %) had a total of 4 events, 1.0 episode per affected patient

Improvement in aggregate DS3 scores occurred despite broad ERT dose ranges within each patient sub-group. Regardless of DS3 severity category, the mean initial ERT dose was approximately 45 units/kg/Q2W compared to 35 units/kg/Q2W after 5 years (Fig. 3) and there were no significant differences in mean cumulative ERT dose at years 1, 2 and 5 between patients who had MCII and those who did not (Table 6). For those patients who attained MCII after 5 years but not at year 1, the cumulative ERT dose during year 1 was not substantially lower than in those patients who improved more rapidly.

Figure 3 shows the longitudinal changes in mean (SD) DS3 scores for up to 22 years. Despite considerable evaluable patient attrition after 10 years, mean DS3 scores are relatively static after the first 5 years. However, despite the overall favorable impression of response to ERT, new episodes of skeletal pathology continue to occur sporadically throughout the 20-year observation period especially, but not exclusively, in the patients with the highest pre-treatment DS3 severity scores. Among the patients in whom the baseline DS3 score exceeded 6.00, 38 of 58 (65.5 %) had a total of 82 new episodes of either AVN, fracture or appearance of lytic lesions while on treatment. 19 patients had multiple new bone events. 73.7 % of the 38 patients with new bone events were splenectomized and 34 (89.5 %) had a pre-treatment history of AVN, fracture or lytic lesions. 10 patients with a positive pre-treatment skeletal history did not develop new bone events while on treatment. Among the moderate severity patients, 15 of 53 (28.3 %) had a total of 19 new bone events. Unlike the marked severity patients, the spleen was intact in 93.3 % and 86.7 % had no pre-treatment record of AVN, fracture or lytic lesions. Seven patients with such a history had no severe skeletal complications after initiation of ERT. Despite the disparity in prevalence of splenectomy and pre-treatment skeletal disease between the marked and moderate patients, the nature of post-treatment bone complications was similar in both sub-groups with fractures accounting for 35-40 % of the total. Among the 22 mild severity patients, 4 (18.2 %) had a total of 4 skeletal events while on ERT. All had intact spleens, none had a record of pre-treatment severe skeletal complications and 3 of 4 had AVN.

**Fig. 4:**
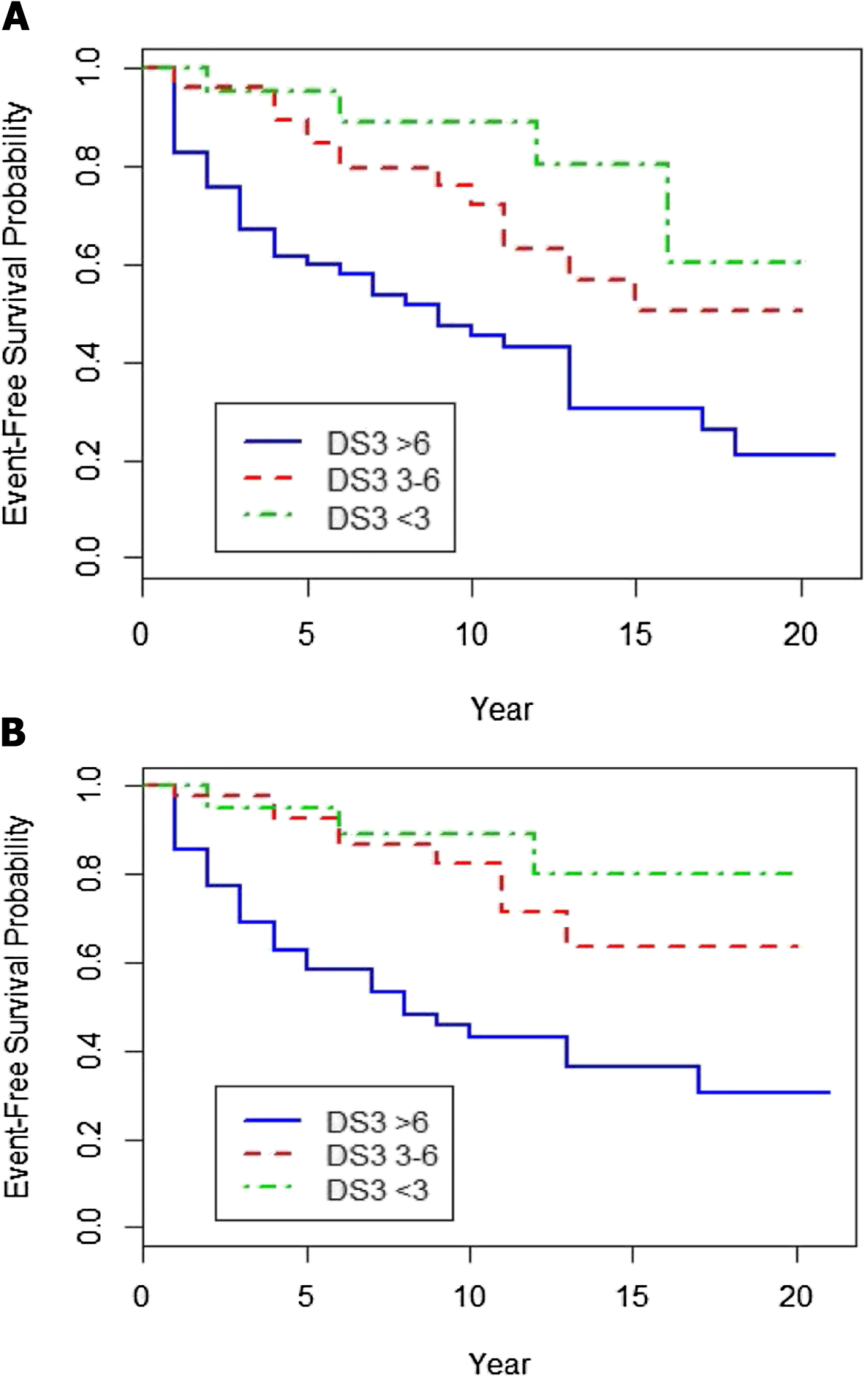
Probabilities, based on baseline DS3 severity status (marked, moderate or mild), of living free of a **a** new episode of avascular necrosis, fracture or lytic bone lesion once GD1 treatment is begun [log-rank test for the difference among groups: *P* = 0.0007], **b** living free of a new episode of avascular necrosis or lytic bone lesion once GD1 treatment is begun (fracture events excluded [log-rank test for the difference among groups: P < 0.0001])

As shown in Fig. 4a, a significant difference in bone event-free survival depended on DS3 severity score grouping prior to initiation of ERT. Despite ERT, patients with pre-treatment marked severity demonstrated a 58 % chance at 10 years and a 70 % chance at 15 years of a severe bone complication. For moderately affected patients, the risk at 10 years is 38 % and 50 % at 15 years and for mild patients, the 10-year probability is 10 % and the 15-year probability is 40 %, although the latter figure may be inaccurate due to small patient numbers. Based on mean (SD) values, no evidence appeared for a significant difference in ERT dose for the year preceding the initial post-treatment bone event between patients with AVN (37.1 [20.8] units/kg/Q2W), lytic lesions (36.7 [24.1] units/kg/Q2W) or fractures (44.8 [25.7] units/kg/Q2W). However, 11 of 38 (28.9 %) patients with either post-treatment AVN or osteolysis were receiving ERT doses less than 20 units/kg/Q2W (3 had treatment interruptions) for one year prior to the event, whereas no patient with a post-treatment fracture had either treatment interruption or a dose less than 20 units/kg/Q2W.

With aging, GD1 patients may have an increased risk for fracture that is at least partly non-GD dependent, whereas, in the absence of concurrent known predisposing factors (corticosteroid use, alcoholism, hyperlipidemia, collagen vascular disease, malignancy), AVN and lytic bone lesions are rare in the general adult population [19, 20] and most likely directly attributable to GD1 pathophysiology. Among the patients who suffered new bone events while on ERT, those with AVN (*N* = 29; mean [SD] age 47.1 [20.1] years) and lytic lesions (*N* = 9; mean age 43.3 [15.3] years) were significantly younger than those with fractures (*N* = 19; mean age 65.6 [10.4] years) (*P* = 0.0001). However, even when fracture events are excluded, ERT-treated patients, especially but not solely those with pre-treatment DS3 scores >6.00, continue at risk for new episodes of AVN and osteolysis irrespective of ERT dose, suggesting that ERT alone is sometimes insufficient for achieving a complete remission of all GD1 manifestations (Fig. 4b).

## Discussion

The GD1-DS3 was devised to provide physicians and allied health care providers with a useful and easily applicable quantitative instrument to complement clinical judgment when assessing and monitoring adult patients with GD1 regardless of treatment status. Referring to the GD1-DS3 as a severity score is somewhat misleading as severity of an illness is properly defined in terms of outcomes such as mortality, morbidity, prevention of end organ damage and reversibility, none of which are yet known to be forecast by DS3. Rather, the GD1-DS3 is a composite measure of disease burden. However, with that caveat and in keeping with earlier literature, we will continue to use “severity” in this discussion.

GD1-DS3 improves upon older scoring systems such as the Zimran severity score index (SSI) [10] by incorporating current standard recommended methods for assessing organomegaly, bone marrow infiltration and bone mineral density. Because published experience with the GD1-DS3 has been rudimentary, we undertook this study of a large, heterogeneous GD1 population to further investigate the value of DS3 for quantitating extent of disease and for monitoring response to treatment. Our results conform to and amplify previous reports addressing these areas and should encourage broader use of DS3 scoring when evaluating the status of adult patients with GD1.

Because of the US/Canada multi-site study design, our patient cohort is representatively diverse in terms of genotype and ethnicity. Based on demographic and GD1 prevalence estimates, the proportion of Ashkenazi Jews in our study is that expected for US/Canada and the genotype distribution is similar to that reported by ICGG in 2013 for 2335 North American GD patients (ICGG 2014 annual report). By restricting this analysis to treated patients only, we assume most N370S homozygous patients with null to mild severity phenotypes are filtered out so that the results from our patient cohort should be applicable to GD1 patients worldwide despite the different genotype profiles. 83 % of our study patients had pre-treatment DS3 scores in either the moderate or marked severity range.

As hypothesized, DS3 scores were consistent with traditional surrogate indicators of GD1 severity. On average, scores were significantly higher for genotype groupings commonly believed to be associated with greater disease severity (N370S/L444P, N370S/other, other/other) than for N370S/N370S [21, 22]. A higher than expected average DS3 score for N370S homozygote patients reflects selection bias towards clinically more severe phenotypes [23]. The mean DS3 score in 18 N370S homozygotes from the untreated patients in our patient population excluded from this analysis (2.16 [1.40]) is significantly lower than the score of 4.33 (2.30) in the treated patients (*P* = 0.0004).

Baseline DS3 scores were significantly greater in patients with a history of pre-treatment splenectomy. Splenectomy in untreated GD1 patients is known to increase risk for AVN [24], liver fibrosis [25], pulmonary hypertension [26] and possibly malignancies [27, 28]. With the exception of AVN, none of our patients presented with these complications prior to inception of ERT. Our results also confirm previous reports indicating a dramatic decrease in the need for therapeutic splenectomy in GD1 patients since the advent of ERT in 1991 [9, 27]. AVN, fractures and osteolytic lesions are traditional indicators of GD1 severity now used in modeling analyses to define stages of GD1 progression [29]. As anticipated, high baseline DS3 scores were significantly associated with a history of pre-treatment severe bone complications. The cumulative incidence of pre-treatment bone events from the date of GD1 diagnosis was 0.14 at ten years and 0.23 at 20 years. This experience closely conforms to that recently reported in a cohort of symptomatic Dutch patients and in the French GD Registry [29, 30].

When coincident with GD1 signs and symptoms, age at diagnosis is a commonly invoked surrogate for disease severity and is a component of the Zimran SSI [10]. In 247 French GD1 patients, age at diagnosis ≤15 years was associated with an increased risk for bone events [30]. Because nearly all the DS3 study patients were diagnosed due the presence of GD1 manifestations rather than by family screening, we postulated that they were likely symptomatic at or prior to date of diagnosis and that a younger age at diagnosis should be associated with a higher pre-treatment DS3 score. Indeed, baseline DS3 scores were significantly higher in patients who were diagnosed before age 18 years than in those diagnosed when older and patients who were categorized as having marked severity disease were significantly younger when diagnosed than those with milder disease scores. However, because age at diagnosis is not solely dependent on onset of clinical GD1 manifestations but is also subject to non-biological determinants such as diagnostic delay, the correlations between age of diagnosis and DS3 severity scores were relatively weak. Although we anticipated a strong direct relationship between baseline DS3 score and years from diagnosis to start of treatment, the correlation was also weak although statistically significant. This confirms repeated observations that progression in adult GD1 patients is stochastic rather than linear and, in particular, pre-treatment bone complications that are heavily responsible for increases in the DS3 score usually occur infrequently and sporadically even over 20-25 untreated years [29–31]. In 24 GD1 sibling groups in whom treatment decisions conformed to relatively uniform guidelines, Amato et al. [32] confirmed the hypothesis that the age at which ERT is begun is a rough surrogate for disease severity. We were unable to demonstrate this relationship in our study because, for many of our patients, age at start of treatment was primarily dictated by the advent of ERT in 1991 rather than by proximal GD1 complications. Furthermore, even for those patients diagnosed after 1991 in whom treatment generally commenced within 0-2 years of diagnosis, heterogeneous criteria for starting ERT thwarted our ability to demonstrate a relationship between age at initiation of ERT and clinical phenotype.

ERT was sometimes initiated at locations other than at the investigative sites and dosing was not uniform. For the entire patient cohort, the mean initial dose of approximately 45 units/kg/Q2W is very close to that reported by ICGG for North America and globally (ICGG 2014 annual report). The proportion of patients in whom ERT was started at ≤15 units/kg/Q2W (12.1 %) is also similar to the global and North American ICGG populations. In many parts of the world, limited health care resources mandate the initial use of lower ERT doses with an option to increase the dose should the patient response be suboptimal. With the exception of Toronto, Canada, there were no such dose limitations at the North American study sites. In 2005, the ICCG US Regional Coordinators recommended that “the initial ERT dose should be determined in the context of the existing severity of disease and the likelihood for continued, progressive or new onset complications…. Lower risk adults may begin treatment at 30 to 45 units/kg every 2 weeks” [33]. Although many of our study patients began treatment years before publication of these guidelines, based on the baseline DS3 scores, we found little evidence that initial dosing decisions were driven by assessment of patient severity.

The observed serial changes in DS3 scores and transitions in GD1 health states (mild, moderate, marked) further demonstrate not only the short-term and longer-term effectiveness of ERT for reversing and controlling common GD1 manifestations but also reveal some of its limitations. In that significant improvement in DS3 scores is largely confined to the first 5 years from inception of treatment followed by a 5-20 year plateau, our findings mirror earlier reports that are more narrowly concentrated than the composite DS3 score on hematological and visceral responses or on changes in surrogate biomarkers such as serum ferritin and chitotriosidase [34–37]. However, the DS3 methodology may be insufficiently sensitive to reflect changes in manifestations that respond relatively slowly to ERT such as bone mineral density [38]. For example, a clinically significant improvement in lumbar Z-score from -2.4 to -1.1 would generate no change in the DS3 score. Tukan et al. discuss the advantage of using absolute BMD values rather than T or Z scores for assessing BMD response in patients with GD1 [39]. A better alternative might be to calculate the DS3 DEXA component using the FRAX^©^ 10 year fracture risk score which corrects for geography, DEXA machinery, body mass index, gender, race, age, smoking and alcohol intake and prior history of fracture. However, an MCID for fracture risk is yet to be determined [40].

Despite evidence that the magnitude and rate of quantitative change in individual response parameters is ERT dose dependent [36, 41], we were unable to establish any relationship between either initial or cumulative ERT dose and outcomes as measured by DS3 scores. A similarly unpredictable association between ERT dosing and composite short-term outcomes has also been reported when achievement of therapeutic goals was the study endpoint [5, 42]. Consequently, we conclude that DS3 scoring will not contribute to resolving the conundrum of ERT dosing, which, because of complex individual variation, socioeconomic considerations and insufficient knowledge of long-term outcomes, likely will remain empirical rather than formulaic for the foreseeable future [39, 42].

The likelihood of experiencing improved DS3 scores with ERT was not affected by baseline DS3 severity, genotype (see also [42, 43]) and minimally, if at all, by the length of time between GD1 diagnosis and initiation of treatment. As previously shown with regard to visceral disease [44], the greatest improvements in total DS3 score and the majority of clinically important responses were recorded in patients who began treatment when markedly affected. Nevertheless, the majority of these individuals and about one third of the patients who were moderately affected at baseline continued to have evidence of residual disease of at least moderate severity after 5 years of treatment up to 15-20 years. This finding is consistent with other literature showing that after 4 years of ERT at variable doses, splenomegaly 8-fold or greater persists in 20-40 % of ERT treated patients, thrombocytopenia in non-splenectomized patients in 20-30 %, bone pain in 30-40 % and osteopenia in 30 % or higher depending on patient age [5, 6, 42, 45]. After 10 years on imiglucerase, 41 of 93 patients with moderate or severe splenomegaly at baseline still had spleen volumes 5-15 times normal, and 15 of 84 patients with moderate or marked pre-treatment hepatomegaly continued to have liver volumes 1.25-2.5 times normal. In 38 %, the platelet count was less than 120,000/μL and 30 % had continuing bone pain [35]. In a recent report from the Netherlands, of 28 non-splenectomized GD1 patients without bone complications but with other signs and symptoms sufficient to justify initiation of ERT, 25 % did not achieve “recovery” (roughly equivalent to DS3 mild severity) through 10 years [29]. The prognostic importance of residual laboratory and imaging abnormalities relative to patient-reported health assessments and with respect to long-term outcomes such as GD1-associated Parkinsonism and malignancies in treated GD1 patients is still undetermined [46] and will be a focus of continued prospective evaluation in our study patients.

New AVN and lytic lesions in previously unaffected sites in ERT-treated patients are another manifestation of incomplete response that may be mechanistically different from the persistent disease manifestations discussed above. Residual hepatosplenomegaly, hypersplenic thrombocytopenia and bone pain are likely indications of irreversible end-organ damage (infarction, fibrosis, osteoarthritis) rather than of treatment resistance. Fractures may have complex etiologies not necessarily related to GD1. Among the patients with marked baseline disease, it is not surprising that nearly half suffered new AVN or lytic lesions while on ERT given multiple risk factors including high prevalence of splenectomy, frequent history of pre-treatment bone events and long interval between diagnosis and treatment [24]. On the other hand, the observation that 12 of 75 (16 %) patients with baseline disease scored as moderate or mild, had AVN or lytic lesions despite continued treatment is more novel. Although delayed start of ERT may have been a contributory factor in 8 patients, only one of 12 had a history of splenectomy and pre-treatment bone events occurred in only 2 of 12. The inability of ERT to completely prevent bone complications even in patients with mild phenotypes may represent a therapeutic class limitation and a justification for research and development of new pharmacologic treatments for GD.

## Conclusions

DS3 is an effective tool for assessing pre-treatment disease burden in GD1 and for monitoring response to therapy. It is consistent with indirect indicators of GD1 severity such as genotype and early age of diagnosis and symptom onset. ERT is associated with clinically important improvement in DS3 scores in patients with high severity scores. Nevertheless, even with sustained improvement in DS3 scores, GD1 patients on ERT, especially those with splenectomy and pre-treatment bone pathology are at continual risk for emerging major bone complications. The likelihood of ERT-emergent bone complications is only partly predicted by either initial or serial DS3 scores and the prognostic value of DS3 scoring for late-onset complications of GD1 such as Parkinsonism, peripheral neuropathy and GD1-associated malignancies remains to be determined. DS3 scoring also needs to be correlated with patient-reported outcomes and used in conjunction with validated GD1-specific patient reporting tools when such instruments become available.
